# Liquid Biopsy Based Circulating Biomarkers in Metastatic Prostate Cancer

**DOI:** 10.3389/fonc.2022.863472

**Published:** 2022-05-20

**Authors:** Eshwari Dathathri, Khrystany T. Isebia, Fikri Abali, Martijn P. Lolkema, John W. M. Martens, Leon W. M. M. Terstappen, Ruchi Bansal

**Affiliations:** ^1^Department of Medical Cell BioPhysics, Faculty of Science and Technology, Technical Medical Center, University of Twente, Enschede, Netherlands; ^2^Erasmus Medical Center Cancer Institute, University Medical Center Rotterdam, Department of Medical Oncology, Rotterdam, Netherlands

**Keywords:** Metastatic prostate cancer, circulating biomarkers, circulating tumor cells (CTC), secretory factors, extracellular vesicles (EVs), prognostic biomarkers

## Abstract

Prostate cancer is the most dominant male malignancy worldwide. The clinical presentation of prostate cancer ranges from localized indolent to rapidly progressing lethal metastatic disease. Despite a decline in death rate over the past years, with the advent of early diagnosis and new treatment options, challenges remain towards the management of metastatic prostate cancer, particularly metastatic castration sensitive prostate cancer (mCSPC) and castration resistant prostate cancer (mCRPC). Current treatments involve a combination of chemotherapy with androgen deprivation therapy and/or androgen receptor signalling inhibitors. However, treatment outcomes are heterogeneous due to significant tumor heterogeneity indicating a need for better prognostic biomarkers to identify patients with poor outcomes. Liquid biopsy has opened a plethora of opportunities from early diagnosis to (personalized) therapeutic disease interventions. In this review, we first provide recent insights about (metastatic) prostate cancer and its current treatment landscape. We highlight recent studies involving various circulating biomarkers such as circulating tumor cells, genetic markers, circulating nucleic acids, extracellular vesicles, tumor-educated platelets, and the secretome from (circulating) tumor cells and tumor microenvironment in metastatic prostate cancer. The comprehensive array of biomarkers can provide a powerful approach to understanding the spectrum of prostate cancer disease and guide in developing improved and personalized treatments for patients.

## 1 Introduction

Prostate cancer (PCa) is the second leading cause of cancer-related death in men (1). Based on GLOBOCAN 2020 estimates, over 1.4 million new cases of PCa and 375,304 deaths were reported worldwide and PCa was the most frequently diagnosed cancer in 112 countries in 2020 (2). Despite a decline in death rates over the past few years, due to early diagnosis and new treatment regimens, PCa remains the most dominant male malignancy worldwide. The 5-year relative survival rate for localized PCa is almost 100%, while for metastatic prostate cancer (mPCa) it is about 30% (3, 4). Patients with metastatic disease thus still have a dismal prognosis; hence there is an unmet need for improving outcome in these patients.

**Figure 1:**
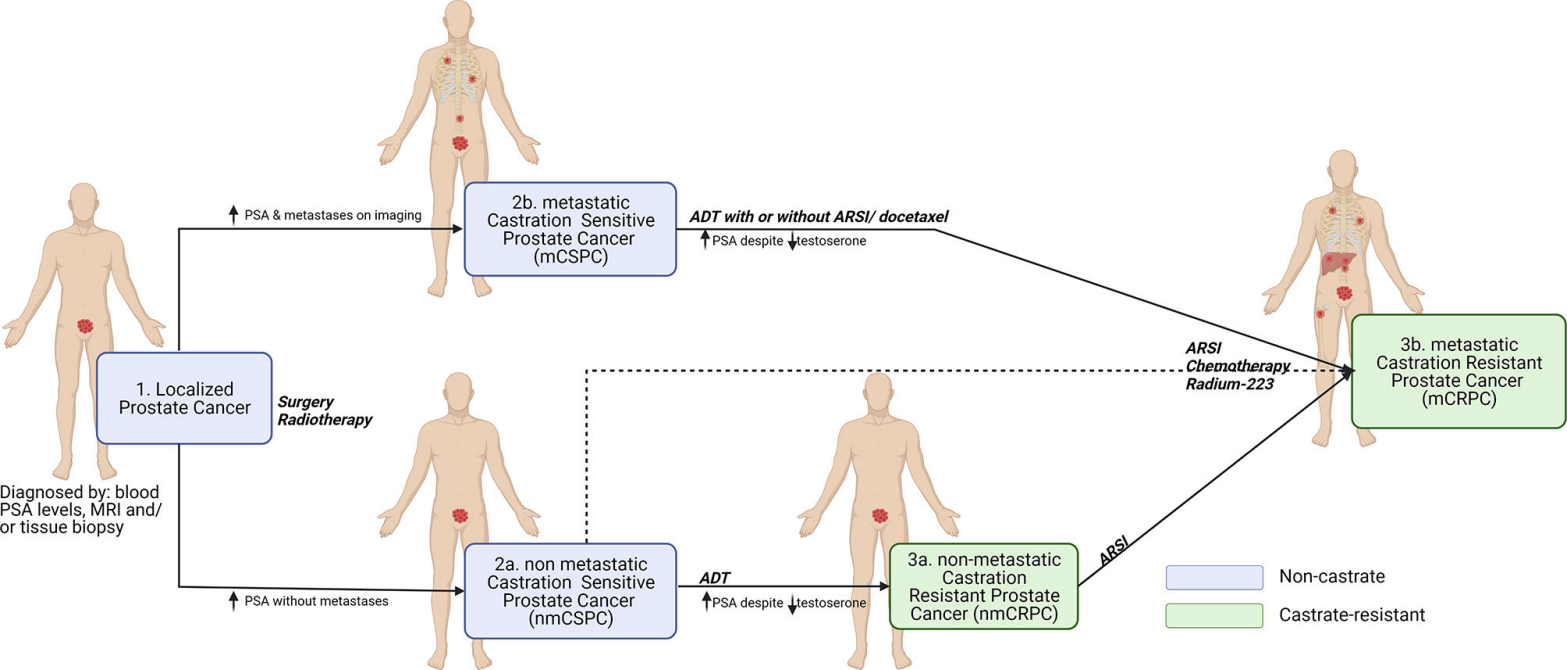
Clinical disease states of prostate cancer. The onset of prostate cancer begins with the localized tumor formation in the prostate glands as diagnosed by blood PSA levels, MRI and/or tissue biopsy (1). However, some patients experience biochemical recurrence and can be diagnosed with high PSA levels with or without metastasis in distant regions of the body (e.g., Bone, lymph nodes, liver, and lungs) leading to non-metastatic castration sensitive prostate cancer nmCSPC (2a) or metastatic castration sensitive prostate cancer mCSPC (2b). Despite treatments with androgen deprivation therapy (ADT) with or without androgen receptor signaling inhibitor (ARSI) or chemotherapy (docetaxel), if the PSA and testosterone levels increase within the castrate range, then the disease is considered to have become (non)metastatic castration resistant prostate cancer (n)mCRPC (3a and 3b). To control the symptoms and to reduce the cancer progression, mCRPC is commonly treated with a combination of ARSI, radiotherapy (Radium-223) and chemotherapy. Created with Biorender.com.

A central feature of prostate cancer is hormone (androgen) responsiveness, whereby castration or androgen deprivation therapy (ADT) causes tumor regression in most of the prostate cancer patients (3, 4). ADT targets the androgen receptor (AR) to inhibit tumor progression as a first line of therapy in patients with hormone sensitive or castration sensitive prostate cancer (HSPC or CSPC). Resistance to castration (or ADT) can result in primary castration resistant prostate cancer (CRPC) or metastatic CRPC (mCRPC) (**Figure 1**). A landmark study, by Huggins and Hodges, in 1941 led to the discovery of castration and/or androgen deprivation as effective treatment(s) for mPCa (5). This early discovery led to the insight that testosterone and AR signaling are key determinants of disease progression. Since 1941, ADT, by surgical and chemical castrations, has been used for the management of patients with advanced and metastatic PCa. The latest findings also show that AR signaling reactivation plays a major role in the emergence of (m)CRPC (6–8).

In the last decade, major progress has been made towards understanding the pathogenesis of metastatic prostate cancer. Consequently, significant advancements in the treatment of metastatic HSPC or CSPC patients have been observed by the inclusion of docetaxel (chemotherapy) and/or AR signaling inhibitors (ARSI), also called novel hormonal therapies (NHTs) such as abiraterone, enzalutamide, and apalutamide. This introduction of chemotherapy and/or ARSIs as adjunct to ADT for most patients showed significantly improved median overall survival (OS) (9). Unfortunately, these advances have come with considerable novel challenges. The trials that were conducted showed significant heterogeneity in patient prognosis and difficulty in choosing/personalizing the treatment. In several trials, patients have been stratified according to their assumed disease load. In these analyses, consistently, chemotherapy seems to render no benefit in patients with so-called low volume disease. Treatment with ARSIs does not seem to depend on the initial volume of disease; however, initial disease load or risk of early treatment failure seem to affect the magnitude of gains, i.e., OS in randomized trials. A review published elsewhere summarized the clinical trials performed in advanced prostate cancer patients including mCSPC and mCRPC, leading to recent drug approvals and discussing optimal treatment selection (10). Improved biological and prognostic stratification of mCSPC patients, therefore, might be helpful to further improve outcomes. In this review, we explore which (circulating) biomarkers are available to improve the prognosis in mPCa patients, and biomarkers that may help to select more personalized treatments for these patients.

## 2 Metastatic Prostate Cancer Biomarkers: PSA and Beyond

Prostate specific antigen (PSA) is the most widely used biomarker for PCa. However, population-based PSA screening remains controversial as this led to considerable false positive results due to poor specificity and thereof overdiagnosis and overtreatment (11, 12). Many men have a raised PSA level without having cancer (high false-positive rate) i.e., due to non-cancerous enlargement or inflammation of the prostate. Conversely, a substantial number of men (15-20%) with a low PSA level (<4 ng/ml) have prostate cancer with advanced Gleason scores (false negative detection) (13, 14). Moreover, PSA fails to distinguish between localized and metastatic PCa (15). Several forms of PSA, i.e., free PSA, or isoforms of PSA, i.e., -2 proPSA, have been evaluated alone or combined for PC screening, i.e., the prostate health index (PHI = (-2 proPSA/free PSA) x total PSA^1/2^) that improved the accuracy of PCa predictors at biopsy (16, 17). Other clinically validated (serum and urine) biomarkers, when combined with PSA, showed improved diagnostic accuracy include Prostate cancer antigen 3 (PCA3), Mi-Prostate Score (MiPS), 4K score, epiCaPture, and STHLM3 (15).

**Figure 2:**
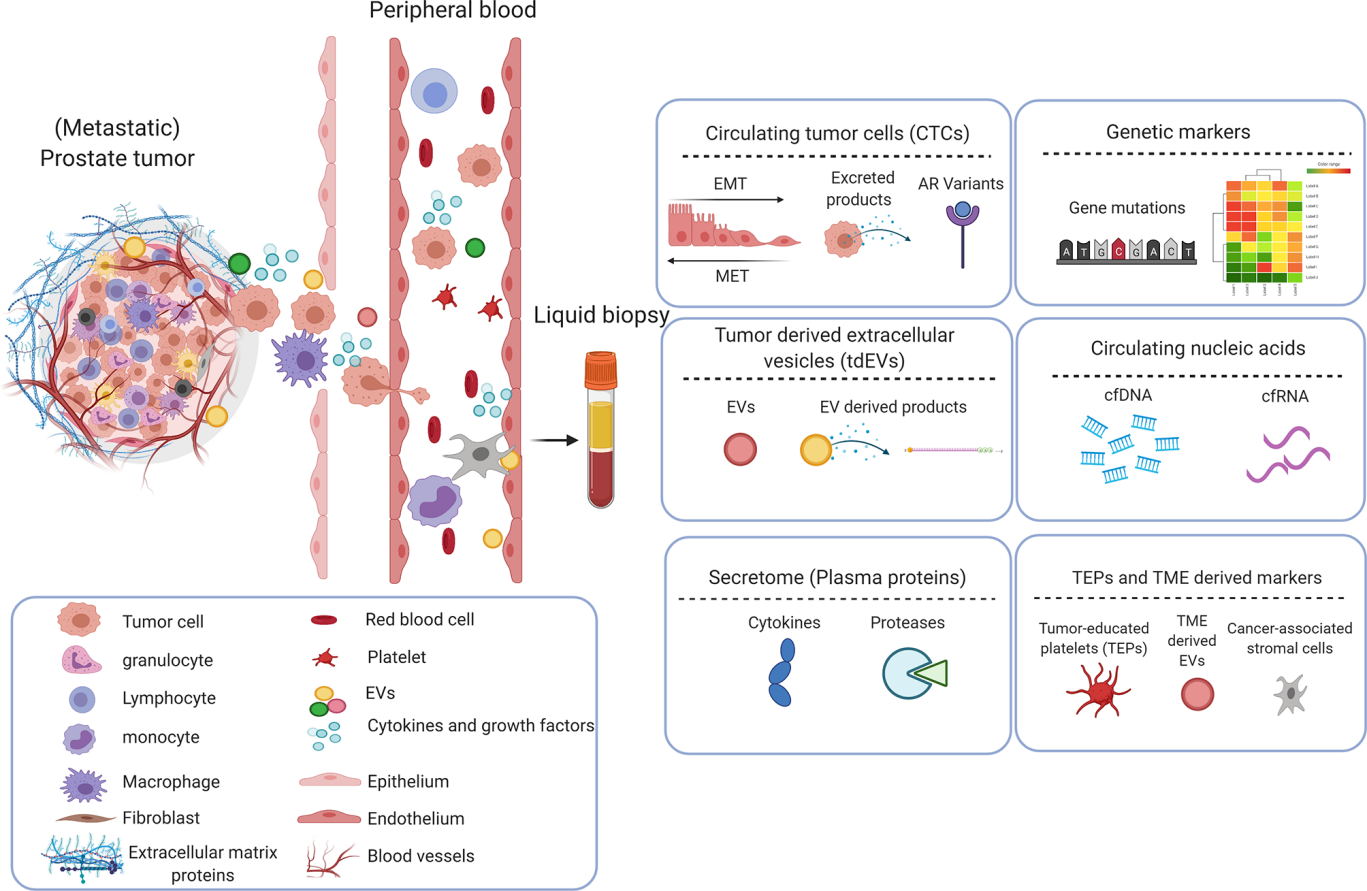
Circulating biomarkers in prostate cancer. Primary prostate cancer metastasizes when tumor cells and cellular components break away and enter circulation (vasculature or lymphatics), travel to distant sites and form secondary tumor(s). These circulating tumor components provide an insight into the phenotypic and genotypic properties of the tumor, can act as a prognostic and predictive tool in determining the outcome of treatments. Circulating tumor cells (CTCs), extracellular vesicles (EVs), secretome (plasma proteins), genetic markers, circulating nucleic acids and other cells i.e., cancer-associated stromal cells or tumor-educated platelets (TEPs) in circulation, have been identified as potential biomarkers and is explored to further understand the clinical progression of prostate cancer and to aid development/optimization of treatments. Created with Biorender.com.

Despite the emergence of other markers, PSA remains one of the prognostic markers for assessment of OS in mPCa patients. A study (in accordance with the Southwest Oncology Group, SWOG 9346 trial) concluded that 7-month PSA ≤ 0.2ng/mL is prognostic for longer OS of mCSPC patients on ADT (with or without docetaxel) (18, 19). However, the availability of a prognostic biomarker that could detect clinical benefit (or lack thereof) earlier than current long-term end points such as overall survival would aid in trial design and drug development. To facilitate the identification of (novel) biomarkers, liquid biopsy is a promising technique to screen body fluids that reflect disease progression and treatment response (20). Liquid biopsy analytes include circulating tumor cells (CTCs), extracellular vesicles (EVs), circulating cell-free nucleic acids [cell-free DNA (cfDNA), circulating tumor DNA (ctDNA), cell-free RNA (cfRNA) and circulating tumor DNA (ctRNA)], genetic markers, secretome (plasma proteins), Tumor educated platelets (TEPs) and other circulating cells in tumor microenvironment (TME) have been investigated as mPCa biomarkers (**Figure 2** and **Table 1**). Besides circulating biomarkers, recognizing the distinct genetic features within metastatic prostate cancer, through testing for AR splice variants (AR-Vs) or tumor suppressor genes (TSGs), might also lead to prognostic and predictive biomarkers for precision medicine.

**Table 1:** Summary of circulating biomarkers used in clinical studies and trials.

Biomarker	Population	Treatment	Outcome	Ref.
Circulating tumor cells (CTCs)	170 mCRPC patients with ≥ 5 CTCs in 7.5 mL of blood	Androgen signaling inhibitors (ARSI)	Patients with high CTC heterogeneity (phenotypic) showed shorter OS and progression-free survival (PFS). CTC heterogeneity was subjective to change with ARSI treatment.	(21)
	191 mCRPC patients	128 pre-ARSI and 63 pre-taxane	mCRPC patients with AR-V7 CTCs showed shorted OS, PFS and resistance to posttherapy PSA changes before ARSI compared to those without AR-V7 CTCs.	(22)
	29 mPCa patients and 25 non-metastatic PCa	–	Glucose metabolic (GM)-positive CTCs improved marker panel compared to EMT-CTC phenotypes.	(23)
Tumor derived extracellular vesicles (tdEVs)	84 mCRPC patients	–	Unfavorable patient groups (>5 CTCs and >105 tdEVs) associated with poor OS.	(24, 25)
	89 patients with different stages of PCa; 35 CRPC patients	–	Exosomal AR-V7 mRNA associated with lower hormone levels and poorer prognosis in CRPC.	(26)
Circulating nucleic acids	122 mCSPC patients, 112 localized PCa and 34 healthy subjects	–	Increased cfDNA plasma concentrations in mCSPC compared to localized PCa and healthy subjects.	(E. 27)
	67 mCRPC patients	ARSI or Taxane therapy	AR gain and AR-V+ expression correlated with poor prognosis, was associated with shorter OS and PFS in both ARSI-treated and chemotherapy-treated cohorts.	(28)
	53 mCRPC patients	ADT	High ctDNA predictive of ADT failure	(29)
	202 mCRPC patients	ARSI	BRCA2 and ATM defects in ctDNA associated with poor clinical outcome. Somatic changes in TP53 were associated with resistance.	(30)
	125 mCRPC patients 25 mCRPC patient	Prednisone or Enzalutamide	High ctDNA associated with presence of bone metastasis, increased levels of PSA and lactate dehydrogenase.	(31)
Secretome (Plasma proteins)	44 mCRPC patients	ARSI	Higher baseline levels of IL-6 in treatment-resistant patients compared to treatment-sensitive patients.	(32)
	233 mCSPC patients	ADT monotherapy ADT + Docetaxel	Higher IL-8 levels in docetaxel-treated patients compared to ADT monotherapy. Higher IL-8 levels prognostic for poor OS, shorter time to CRPC, independent of docetaxel use and metastatic burden.	(33)
	44 mCRPC patients	ARSI	Higher baseline levels of IL-10 in ARSI-resistant patients compared to ARSI-sensitive patients.	(32)
	215 PCa patients	–	Higher MMP-2 expression in CTCs and DTCs of patients with bone metastasis.	(34)
	93 localized PCa and 13 mPCa patients	–	MMP-7 serum concentration higher in bone metastatic patients compared to localized PCa.	(35)
	7 mCRPC patients	–	Treatment-responsive patients showed lower MMP-2 and MMP-7 levels compared to patients with metastasis (bone and lymph node).	(36)
Tumor educated platelets (TEPs)	50 mCRPC patients	Abiraterone and docetaxel	*KLK3*, *FOLH1*, *NPY* transcripts in TEPs indicated poor OS and accurately predicted outcome after abiraterone therapy.	(37)

mCRPC, metastatic castration resistant prostate cancer; ARSI, androgen signaling inhibitors; AR-V7, Androgen receptor variant 7; CTCs, circulating tumor cells; OS, overall survival; PFS, progression free survival; GM, Glucose metabolic; PSA, prostate specific antigen; EMT, epithelial to mesenchymal transition; CfDNA, cell free DNA; mCSPC, metastatic castration sensitive prostate cancer; CtDNA, circulating tumor DNA; ADT, androgen deprivation therapy; tdEVs, tumor derived extracellular vesicles; TEPs, tumor educated platelets; KLK3, Kallikrein Related Peptidase 3; FOLH1, Folate Hydrolase 1; NYP, neuropeptide-Y; IL, interleukins; DTCs, disseminated tumor cells; MMP, matrix metalloprotease.

### 2.1 Circulating Tumor Cells

**Figure 3:**
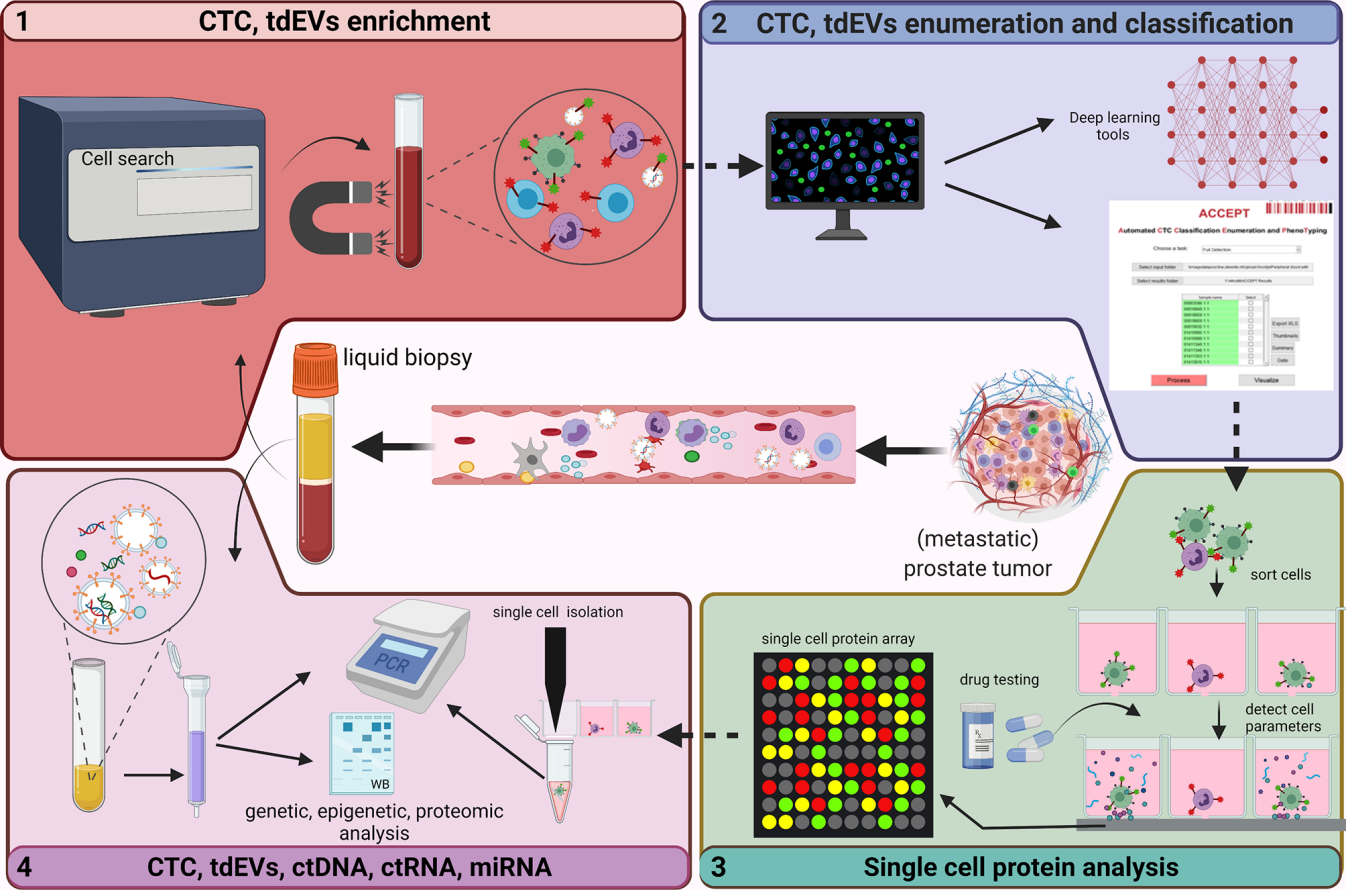
Analysis of circulating biomarkers. This figure depicts the technologies (developed and used at the Medical Cell BioPhysics Department at the University of Twente) that are generally used to identify/analyze circulating biomarkers in liquid biopsy. The FDA approved CellSearch (immunomagnetic enrichment) is the gold standard and is currently used for the CTC and tdEV enrichment from prostate cancer patient diagnostic leukapheresis (DLA) sample (1). An in-house developed tool - Automated CTC Classification Enumeration and PhenoTyping (ACCEPT) - is used to classify and enumerate the CTCs and tdEVs to better understand the morphology and phenotypic heterogeniety. Other deep learning tools such as Deep or Recurrent Convolutional Neural Networks (DCNN or RCNN) based algorithms were developed with improved segmentations models for automated and bias-free CTC enumeration (2). Enriched CTCs are further sorted into single cells using microwell arrays, analyzed for protein secretions in response to treatments, after which CTCs of interest are isolated (3). Omics profiling of selected CTCs and other circulatory markers including tdEVs, ctDNA, ctRNA and miRNA using polymerase chain reaction (PCR) and low-pass whole genome sequencing (LP-WGS) is performed to identify aberrations and analyze tumor heterogenity and aggressiveness (4). Created with Biorender.com.

CTCs are tumor cells disseminated from primary and/or metastatic tumor sites that circulate in the vasculature with potential for distant seeding. Compared with traditional biopsies, CTCs are considered to better reflect inter- and intra-tumor heterogeneity. Although present at relatively low frequencies, CTC presence is associated with poor prognosis; their rise or decline is a strong and early predictor of treatment response, and their characterization can be used to determine treatment options (38). CTC enumeration using Food and Drug administration (FDA)-cleared CellSearch system (**Figure 3**) is the most frequently used system to assess prognosis and to determine early response to therapy in various metastatic carcinomas including metastatic prostate cancer (39–43). While CellSearch is useful to enumerate the CTCs, a major limitation is that it captures only Epithelial cell adhesion molecule (EpCAM) positive cells, and EpCAM-negative CTCs cannot be isolated by this system. The CTC yield also shows low viability and cannot be cultured for long periods (44). To overcome this limitation, multiple microfluidic technologies have been explored in the last two decades (45). A recent assay developed by Epic Sciences, known as the AR-V7LB, counts both EpCAM-positive and EpCAM-negative cells suggesting the epithelial to mesenchymal transition (EMT) (46, 47). A recent study showed that it was a useful platform in detecting AR-V7 in CTCs of mCRPC patients starting second generation treatment with ARSI, with a strong correlation to the CTC counts from the CellSearch platform (48).

As mentioned before, CTCs can be derived from epithelial cells, mesenchymal cells, and/or can be of hybrid phenotype. EMT is the characteristic feature of CTCs whereby epithelial cells gain the migratory properties of mesenchymal cells and the process is considered to play an important role in metastasis (49). Likewise, disseminated cells recover epithelial properties for rapid colonization through the mesenchymal to epithelial transition (MET). Related CTC phenotypes including E-CTCs (epithelial), M-CTCs (mesenchymal) and H-CTCs (hybrid) act as promising biomarkers. E-CTCs can be characterized by epithelial markers such as EpCAM, E-cadherin, Prostate specific membrane antigen (PSMA), cytokeratin (CK); M-CTCs express mesenchymal markers (Vimentin, Twist), and H-CTCs co-express epithelial and mesenchymal markers e.g. cytokeratin and vimentin as molecular markers for EMT (49). In an observational study, the involvement of survivin, an inhibitor of apoptosis, highly expressed in mCRPC and associated with poor clinical outcome in CTCs has been suggested and the study showed that siRNA-mediated survivin-knockout inhibited EMT and invasiveness of CTCs and DU145, an AR-independent metastatic PCa cell line (23). Intriguingly, another study suggested that hypermetabolic GM^+^ CTCs expressing glucose metabolic markers (PGK1/G6PD) as a promising biomarker, compared to the EMT CTCs subtypes, for diagnosis of mPCa. This study suggested a triple tPSA–Gleason–GM^+^CTCs marker panel with an improved AUC of 0.904, compared to the tPSA–Gleason–H-CTCs marker panel (0.874) (50). Thus, besides enumeration, CTCs phenotyping and comprehensive omics analysis (**Figure 3**) can improve prognostication or treatment prediction of mPCa.

In mCRPC patients, the presence of ≥5 CTCs in 7.5mL of blood predicts an unfavorable prognosis, whereas a post-treatment drop in CTC count predicts an improved prognosis (42). A multicentric study replicated these findings using an open-source Automated CTC Classification Enumeration and PhenoTyping (ACCEPT) software (**Figure 3**) for the prognostication of mCRPC patients (21). ACCEPT is a useful tool to detect and enumerate fluorescently labeled CTCs (and EVs) where classification into different subsets is based on linear gates applied to extract measurements of objects identified by a multiscale segmentation model. However, the use of linear gates causes segmentation of objects that lie very close to one another as one event, resulting in false positives that need to be eliminated during the manual review of thumbnails (51). Hence, there exists a need to develop fully automated and robust techniques to better enumerate CTCs. Deep and recurrent convolutional neural networks (DCNN or RCNN) have been developed and are currently used with improved segmentation that effectively classify and allow bias-free enumeration of CTCs (51–53). Moreover, few studies in mCSPC patients suggest that baseline CTC count is highly predictive of 7-month PSA response and 2-year progression-free survival (PFS), and that CTC count correlate with OS and PFS (54, 55). Besides CTC count, CTC phenotypic heterogeneity can help in making informed decisions about the therapy. It was shown that low CTC phenotypic heterogeneity was associated with better OS in ARSI-treated patients, whereas high heterogeneity was associated with improved OS in patients treated with taxanes (22).

Several genetic aberrations associated with castration resistance under anti-hormonal therapy include the amplification of AR (and/or the respective upstream enhancer) and alterations with the ligand binding site, altering ligand-preferences, or even enabling a constitutionally active isoform. Besides, AR alternative splicing isoforms (AR-v’s) are generated as one of the mechanisms for PCa transition to castration resistance. AR splice variants (AR-V1, 3, 7 and 9), is a phenomenon first reported in 2004, which recently earned renewed clinical relevance due to its role as a dynamic marker throughout disease progression and treatment-induced selective (clonal) pressure (56, 57). Among AR-Vs, the most frequently expressed and biologically significant is AR-V7, a transcript encoding a variant of AR that lack the ligand-binding domain and thereby functions as a constitutionally active nuclear transcription factor (58–60). Scher et al. observed patients with an increasing number of AR-V7 positive CTCs ranging from 3% by first line of therapy to 31% by third or greater line of therapy. Furthermore, it was suggested that patients with AR-V7 positive CTCs were more resistant to ARSI compared to treatments with taxanes, that resulted in longer OS in such patients (61). Moreover, whole blood analysis of CRPC patients revealed higher expression of AR-Vs (68% for AR-V7 and 32% ARv^567es^) in hormone-treated patients compared to the hormone-naïve (62). These studies suggest AR-V7 in PCa as a potential negative predictive biomarker for guiding AR-directed hormonal treatments for CRPC patients. Besides, AR-V3 that also lack the ligand binding domain, cell context-dependent variants like AR-V1 and AR-V9, which are conditionally activated variants have been described and could all play a role in progression to castration resistance and hence a clinical role (63–65).

### 2.2 Genetic Biomarkers

PCa is characterized by a wide variety of genomic aberrations encompassing copy number alterations, genomic mutations such as single-nucleotide variants (SNVs), insertions–deletions (indels) and multi-nucleotide variants (MNVs), and large-scale structural rearrangements. The active mutational mechanisms, mutation rate, and mutational landscape and drivers alter throughout disease progression and (treatment-driven) somatic evolution and clonal dynamics.

Within primary PCa, driver-gene analysis revealed enrichment or recurrence of mutations or copy-number alterations within *SPOP, FOXA1, IDH1, TP53, PTEN, PIK3CA, BRAF, CTNNB1, HRAS, MED12, ATM, CDKN1B, RB1, NKX3-1, AKT1, ZMYM3, KMT2C, KMT2D, ZNF770, CHD1, BRCA2, CDK12* as reported by The Cancer Genome Atlas (TCGA) consortium (66). Additionally, recurrent genomic gains of chromosome 7 and 8q and heterozygous losses of 8p, 13q, 16q, and 18 were often observed within PCa (66).

Compared to another large-scale sequencing study focusing on mCRPC (67), the mutational landscape harbored an overall greater mutational burden and incidence of large-scale structural variants compared to primary PCa. However, the inventory of driver genes remains similar, with the marked exception of (treatment-induced) mutations on AR. The following genes were enriched with mutations compared to primary PCa: *PRAD; AR, TP53, MYC, ZMYM3, PTEN, PTPRD, ZFP36L2, ADAM15, MARCOD2, BRIP1, APC, KMT2C, CCAR2, NKX3-1, C8orf58*, and *RYBP*. Somatic alterations influencing the expression or function of AR have been reported as one of the main driving forces of castration resistance. Recurrent amplifications of AR and/or its upstream enhancer coupled with splicing aberrations (ARVs) and treatment-related hotspot mutations have been reported previously.

Tumor suppressor genes (TSGs) such as *RB1*, *TP53* and *PTEN* are among the most frequently altered genes in PCa whereby co-operative functional loss of these TSGs has been associated with poor prognosis. Hamid and colleagues found increased TSG alterations (mono-/bi-allelic loss) in mPCa, 63% in mCSPC and 92% in mCRPC versus 39% in localized CSPC. Moreover, two or more TSG alterations were more frequent in mCRPC (73%) compared to mCSPC (28%) and localized CSPC (11%). Finally, it was concluded that TSG variants were linked to early relapse and worse outcomes in the CSPC patient cohort (68). Studies have also revealed a relationship between genetic alterations and disease volume. In the STAMPEDE trial on mCSPC patients, frequent alterations in the *PTEN* in high-and low-volume disease were observed, while *TP53* alterations were found in low-volume disease (69). Stopsack and colleagues, found an association of *SPOP* alterations with improved prognosis, while aberrations in AR, cell cycle and *TP53* were associated with worse prognosis (70). Moreover, *RB1* loss was associated with poor prognosis in mCRPC patients (71 Apart from these TSGs, *BRCA2* (PROREPAIR-B study) and *CDK12* mutations have been associated with increased aggressiveness and metastases, short OS time and poor response to first-line therapy (3, 72).

Next to altered genes and mutations, PCa commonly harbors gene fusions and several solid tumors that demonstrate a high frequency of recurrent gene fusions (73, 74). One of the gene fusions is the enigmatic *TMPRSS2-ERG* fusion observed in 50-70% of all PCa cases, yet incidences have been reported to differ among different ethnic and geographical groups (66, 67, 73). *TMPRSS2-ERG* is a fusion between the AR-regulated transmembrane protease serine 2 (*TMPRSS*2) and v-ets erythroblastosis virus E26 oncogene like (*ERG*) genes, and is reported as an early event in PCa initiation (75). Fusion of these genes results in androgen-dependent transcription of *ERG* in prostate cancer cells. The *TMPRSS2-ERG* fusion is the most common fusion event within PCa, however, additional (less frequent) ETS-factor fusions have been reported (76). Due to the high prevalence of *TMPRSS2-ERG*, this somatic event is one of the predominant molecular classification factors and could be used as a promising diagnostic and prognostic marker (75–77). Overall, detection of genetic aberrations in mCSPC and mCRPC might have predictive value for defining treatment landscapes for these patients.

### 2.3 Extracellular Vesicles

Prostate cancer cells release extracellular vesicles (EVs) comprising of apoptotic bodies, microvesicles and exosomes (78). EVs contain bioactive cargo (proteins, nucleic acids, and metabolites), and are increasingly recognized as a pivotal player that play a crucial role in communication between tumor cells and the TME and can also act as prognostic and diagnostic markers. Studies indicated that PCa patients have 4-fold higher levels of nanovesicles expressing both PSA and CD81 (exosomal marker) compared to benign prostate hypertrophy (BPH) patients and healthy individuals where TME acidity seems to regulate the release of PSA-EVs in the blood of PCa patients (79).

Tumor-derived EVs (tdEVs) are also known to regulate osteoclasts and osteoblasts in the bone metastasis of PCa patients (78). A recent study has also indicated that EVs derived from mesenchymal-like prostate cells promote EMT of epithelial-like prostate cancer cells and render resistance to ADT (80). MiR-34a bearing EVs were suggested as a predictive biomarker since it was observed to promote sensitivity to docetaxel by decreasing endogenous B-cell Lymphoma 2 (BCL-2) expression (81). RNA sequencing also revealed other exosomal miRNA such as miR-1290 and miR-375, whose high levels were associated with poor OS in CRPC patients (82). Exosomal CD44v8-10 mRNA copy numbers in EVs were higher in docetaxel-resistant CRPC patients than in docetaxel naïve patients and control men (83). Joncas et al. studied plasma EVs as phenotypic biomarkers in PCa patients with different stages of disease progression in CRPC patients. Authors demonstrated a novel association between high levels of AR-V7 exosomal mRNA (with undetectable androgen levels) and high neutrophil-to-lymphocyte ratio (26). High expression of full-length androgen receptor (AR-FL) was also linked with AR-V7+ CRPC patients and predicted resistance to hormonal therapy (84, 85).

Nanou et al., investigated the clinical relevance of EpCAM+, CK+, DNA-, CD45- tdEVs using the CellSearch system in blood of CRPC patients. The availability of advanced image analysis made it possible to interrogate the images gathered by the CellSearch system for CTC enumeration revealing the presence of several subclasses of CTC and tdEV (51). Using the ACCEPT tool, well-defined tdEVs and CTCs were enumerated in CRPC patients (**Figure 3**), and patients with >5 CTCs and >10^5^ tdEVs were associated with poor OS. Moreover, in the same study, tdEVs showed improved predictive power, regarding sensitivity, and specificity, when compared to CTC count alone (A. 24, 25). The high incidence and superior stability of tdEVs in circulation compared to CTCs, and the tdEVs cargo reflecting tumor heterogeneity make them a promising biomarker in the metastatic setting. However, their small size makes enumeration and characterization more challenging, but with advances in technology this is expected to improve in the near feature.

### 2.4 Cell-Free Nucleic Acids

Cell-free DNA (cfDNA) are DNA fragments that are released into circulation by dying or apoptotic cells in healthy individuals. cfDNA is considered to originate from hematopoietic cells, and in cancer patients, cfDNA constitutes DNA derived from tumor cells (ctDNA) next to those derived from hematopoietic cells. In mCSPC patients, Chen et al., found increased plasma cfDNA concentrations compared to 112 patients with localized disease and 34 healthy subjects. Moreover, more, and shorter sized cfDNA fragments were correlated with an increased risk of localized disease compared to healthy subjects. Although cfDNA was useful in distinguishing between the two groups, cfDNA fragment size showed poor predictive performance due to its low sensitivity and specificity (27).

ctDNA has emerged as a promising biomarker with diagnostic, predictive and prognostic applications in cancer. Genomic analysis of plasma ctDNA has gained attention in recent years and aberrations in ctDNA overlapped substantially with those in tumor tissue, especially with those in bone lesions of mPCa patients (86). Several techniques have been employed for the detection and characterization of ctDNA within the total pool of cfDNA. The sequencing techniques involved can be classified into Sanger sequencing, Pyrosequencing, and next-generation sequencing (NGS). NGS holds an advantage as its sensitivity is 10 times higher compared to the other methods. Hence, it is the most commonly used sequencing technique to detect ctDNA, which is less than 1% of the total cfDNA pool (47). ctDNA can be detected using different types of biomarkers including tumor-specific somatic mutations, copy number variations (CNVs), phasing of histones and methylation patterns (87, 88). Mutant molecules and CNV numbers were consistent with metastatic tissue and the amount of ctDNA reflected poor prognosis (29, 30, 86). In a study by Wyatt et al., next-generation sequencing was performed across 72 genes in 45 cfDNA samples (ctDNA greater than 2% of the cfDNA). The results indicated AR alterations, mutations in SPOP and tumor suppressor genes and alterations in the AR, PI3K and WNT pathways of mCRPC patients who showed disease progression following one line of AR targeted therapy (86). ctDNA is also abundant in most mCSPC patients, providing additional insight into metastatic disease beyond the information diagnosed in primary prostate biopsy. Additionally, higher ctDNA levels were predictive of ADT failure and shorter metastasis-free survival (29–31, 88, 89). In a study, Fettke and Kwan et al. performed integrated sequencing of cfDNA and cfRNA from 67 mCRPC patients and found that AR alterations (AR gain and AR-V expression) correlated with poor prognosis (28). Additional larger randomized studies (with and without a specific treatment) are required to determine the potential of cfDNA, ctDNA, and/or cfRNA as a diagnostic, prognostic, or predictive marker for mPCa.

### 2.5 Secretome

The secretome is a class of proteins that are secreted in the extracellular space and is considered a reservoir of potential biomarkers for cancer (and other diseases). In cancer, the tumor cells (and the TME) produce a secretome with an altered composition compared to their normal state. Evidence from the literature suggests that the tumor secretome plays a vital role in cancer metastasis and progression. Tumor secretome is largely studied using bulk cell approaches, however, this approach fails to identify existing phenotypic and genotypic heterogeneity in specific cells or biomarkers of interest (90). Furthermore, it has become apparent that genetically identical cells can give rise to phenotypic variability (91, 92) indicating that profiling (single-cell) secretome signature is important to gain insights into the tumor heterogeneity, tumor biology, cellular interactive networks, and can be used for individualized diagnosis and therapeutic monitoring.

Single cell omics have gained importance over the recent years as it provides a valuable platform to measure multiple molecules such as DNA, RNA, and proteins secreted from a single cell. One of the major clinical challenges in early diagnosis and designing effective personalized therapeutics has been the lack of adequate technologies to comprehensively characterize inter- and intra-tumor heterogeneity. A recent study developed a novel technology (**Figure 3**) to perform proteomic analysis from single PCa cells. Abali et al., quantified protein secretions from LnCAP and VyCAP cell lines in picograms of PSA produced per cell per day. The effect of different drugs on each cell and their PSA secretions (over a period of 24-72 hours) could be quantified (93). At any time point, the individual cells can be isolated and probed for their molecular composition (94). Single-cell secretome profiling of the tumor allow reconstruction of signaling/communication networks at a systems-level and can provide valuable insights into the origins of tumor heterogeneity, tumor differentiation and evolution, and has the potential to enable the development of more effective personalized medicines for human cancers. The ability to perform such experiments on actual CTCs of mPCa patients could make personalized therapy for mPCa a reality. Availability of a sufficient number of CTC would, however, be a condition to realize this. The use of Diagnostic Leukapheresis enables the harvest of a sufficient number of CTC and as already been successfully accomplished in this disease setting (95). Apart from PSA, other biomarkers in the tumor secretome that can shed light to the complexity of the disease include a variety of cytokines and proteases, however, they lack specificity suggesting a panel of biomarkers would have a greater potential for better prognosis and in predicting therapy response.

#### 2.5.1 Cytokines

Studies indicate that cytokines play a major role in PCa pathogenesis. A study by Pal et al., profiled the levels of cytokines in mPCa patients. The results indicated a decrease in the baseline levels of interleukins (IL) IL-6 and IL-10 and an increase in pro-inflammatory cytokines (IL-5, IL-10, IFN-γ, TNF-α) in ARSI-responsive patients when compared to ARSI-resistant patients (32). IL-8 (CXCL‐8), another pro-inflammatory cytokine released by tumor cells (and macrophages), is significantly higher in cell lines with invasive behavior and metastatic potential such as PC-3 and DU-145 compared to the less invasive LnCAP cells. Studies also found elevated levels of circulating IL-8 in men with bone metastasis compared to those with localized cancer. Harshman et al., studied the impact of serum IL-8 on mCSPC patients in the CHAARTED trial and indicated that at ADT initiation, serum IL-8 levels were elevated and predicted worse OS. The prognostic impact of IL-8 remained independent of metastatic burden, time to metastasis, and docetaxel use, and predicted a short-time to castration resistance (33). IL-23 secreted by myeloid-derived suppressor cells (MDSCs) and activating the pSTAT3-RORγ signaling pathway thereby promoting survival and proliferation of PCa cells, was shown to promote CRPC by activating the AR pathway. Calcinotto et al., observed higher frequency of IL-23-producing MDSCs in tumor biopsies in CRPC compared to CSPC patients, correlating with elevated levels of IL-23 in circulation (96). They further demonstrated that inhibition of IL-23 using IL-23 blocking antibodies restored sensitivity to AD therapy in mice suggesting blocking IL-23 during first-line therapy might reverse resistance to ADT in patients with mPCa (96). These studies show great promise using immune-related circulating biomarkers (cytokines) as predictors of outcome of treatments for patients with mPCa.

#### 2.5.2 Proteases

Aberrantly expressed proteases are excellent candidates for cancer biomarkers, as they play critical roles in various hallmarks of cancer (97). Besides PSA, a protease in the Kallikrein family (KLK3) regulated by androgen signaling, matrix metalloproteases (MMPs) are a family of proteolytic enzymes that are known to degrade extracellular matrix (ECM) and support tumor proliferation and growth as well as migration, and metastasis. Studies have indicated a higher expression of various MMPs (MMP-2, -3, -7, -9, -13, -14, -15 and -26) in metastatic cancer, while MMP-1 expression correlates with early-stage cancer. Among MMPs, MMP-2, -7, -9 and membrane-type (MT)-MMPs are the most extensively studied MMPs in PCa progression (97).

Murray et al., found high MMP-2 expression in CTCs and disseminated tumor cells (DTCs) in mPCa patients. Patients with bone metastases showed an increased expression of HER-2 protein and a resulting increase in MMP-2 expression (34). MMP-3 is also known to promote cancer cell growth and metastasis. Frieling et al., observed high levels of MMP-3 expression in PCa patients with bone metastasis. PCa cells are considered a rich source of MMP-3 in the tumor bone microenvironment. MMP-3 ablation, *in vitro* and *in vivo*, suppressed cancer cells proliferation and reduced bone metastasis (98). High levels of circulating MMP-7 have also been observed in PCa patients with distant metastasis, particularly bone metastasis (35). Significantly elevated mRNA and protein levels of MMP-9, activated by MMP-2 and MMP-3, were seen in malignant compared to benign tumors. In PCa, MMP-2 and MMP-9 are relevant molecular biomarkers that reflect the tumor’s invasive and metastatic potential (97). Dhar et al., analyzed single-cell CTC-secreted MMPs (MMP-1, -2, -7, and -9) in CRPC patients and found that patients responding to treatment and with lower levels of PSA, had lower MMP levels. While CTCs from patients with (bone and lymph node) metastasis showed higher MMP levels (36). These preliminary results indicate a correlation between metastasis, PSA levels, and MMP secretion, thereby establishing a possibility to use MMPs as a prognostic biomarker.

Cathepsins are another family of proteases that not only play an important role in tumor metastasis and progression, but also activate other proteases like proheparanase, urokinase-plasminogen activator (uPA), and MMPs. Cathepsin K (CatK) expression is significantly higher in bone metastasis than in primary PCa and is negative in healthy prostate tissue. Circulating CatK protein expression in conditioned medium was higher in PC-3 and C4-2B (characteristics of bone metastasis) than in LnCAP and PrEC cells. CatK inhibition decreased tumor cell invasiveness, retarded tumor progression, and increased bone density *in-vivo* in mice, supporting an important role of CatK in PCa-induced bone metastasis (43, 99).

### 2.6 Tumor-Educated Platelets

Platelets play an important role during tumorigenesis and cancer metastasis and show altered behavior when exposed to tumors. These so-called TEPs harbor cancer biomarkers that include platelet-derived microparticles, proteins and RNA, and can predict therapeutic response or monitor disease (100). Tjon-Kon-Fat et al., isolated and studied platelet fractions to stratify CRPC patients based on response to therapy. They observed that PCa-associated biomarkers (*KLK3*, *FOLH1*, *NPY* transcripts) in platelets were associated with short OS and enabled prediction of outcome after abiraterone therapy with higher accuracy (37). Although promising, further validation is warranted to determine the potential of TEPs-derived biomarkers for blood-based companion diagnostics, cancer progression (from PCa to mPCa), therapy selection, longitudinal monitoring, and recurrence.

## 3 Biomarkers in Tumor Microenvironment

The tumor microenvironment combining stromal and cellular components surrounding the tumor, can release various biomarkers into circulation, which can be identified in a liquid biopsy. It is paramount to gain insights into the complexity of tumor microenvironment (TME), comprising of immune cells, fibroblasts and endothelial cells, extracellular components (such as proteoglycans, proteins, and glycoproteins) since the communication between TME and tumor cells strongly contribute to tumor progression and metastasis. S. Chen et al., investigated the heterogeneity in infiltrating immune cells and identified a tumor-associated macrophages (TAMs) subset that showed osteoclast-like features (characteristic of bone metastasis). Moreover, elevated expression of *KLK3*, prostate-specific antigen (PSA) gene in T cells (AR-negative) was observed, attributed to extracellular vesicle/exosome-mediated trafficking from the tumor cells to T cells (101). Apart from immune cells, the authors identified three subtypes of cancer-associated fibroblasts (CAFs), surprisingly with depleted expression of *ACTA2* (a common CAF marker in most cancers). *ACTA2* expression correlated with the EMT score suggesting EMT being a possible source for ACTA2-positive CAFs. Moreover, though all CAF subtypes showed angiogenesis-associated features, subtype-specific expression of myofibroblastic, cell adhesion and ECM genes was observed, indicating a shared regulatory network between CAFs and non-fibroblastic lineages in TME (S. 101). Another non-immune component, endothelial cells, are responsible for the recapitulation of tumor heterogeneity within secondary and metastatic sites. Chen et al., identified four EC subtypes, with elevated expression of CAF-related genes, termed activated endothelial cells (aECs). Further analysis revealed interactions of aECs with other TME components suggesting their role in ECM remodeling, hence promoting cancer cell invasion/metastasis, and suppressing immune activation (101, 102).

Prostate tumor-derived EVs are also known to promote a tumor-supportive environment by activating cancer-associated fibroblasts (CAFs), which release miR-409 from EVs and enhance prostate tumorigenesis (103). Docetaxel resistant CRPC derived EVs were also shown to express and secrete Brain4 (BRN4) which promotes the development of neuroendocrine differentiation (NED), leading to an aggressive variant of CRPC (104). Other markers of immunogenicity in the TME have also been shown to be predictive of cancer aggressiveness and treatment resistance. A study characterized heterogeneity of the immune checkpoint expressions of CTCs in mPCa. More than 50% of the CTCs expressed PD-L1 expression in 30% of patients with mHSPC; 20% of patients with mCRPC pre-ARSI and 30% of patients with mCRPC post-ARSI. PD-L2 expression was observed in 20-40% of the patients and B7-H3 expression (>80%) was observed in all patients of all cohorts in the study (105). To develop effective treatments, the complete pathophysiology of mPCa needs to be understood. Hence, identifying biomarkers to study mechanisms behind metastasis, tumors, heterogeneity, and crosstalk within the microenvironment will help in developing tailored treatments for patients.

## 4 Conclusions and Outlook

This review highlights the potential biomarkers in circulation to improve diagnosis and tailor treatments for metastatic prostate cancer patients using liquid biopsies. In the current treatment landscape, developing systemic treatments will become more focused on mCSPC and mCRPC patients. There is a need to start balancing the drive for intensifying treatment, to improve outcomes and to maintain as much quality of life as possible for the men involved. This can be achieved by improving the ability to stratify patients based on their risk of progression, and development of therapy resistance combined with the ability to monitor longitudinally the effects of administered individual (combination) treatments. Liquid biopsy-based strategies are essential to achieve this, since, in metastatic prostate cancer, tumor tissues are often difficult to obtain, and the disease load is often difficult to assess using imaging. Thus, comprehensive analysis of analytes in liquid biopsies will inform us about the biology of the disease, and hence can guide us in making informed decisions about improved and personalized treatments for the patients. Of these analytes, the prognostic biomarkers that were seen to be most effective in the clinics are the CTCs (specificity 67%, sensitivity 69%), tdEVs (69%, 69%), PSA (6-66%, 78-100%) and cfDNA (56%, 88%). These analytes provide useful insight on the overall survival of patients suffering from mCRPC (24, 25, 27). Other biomarkers used in clinics including AR-V7, TMPRSSQ-ERG and secretome proteins also show promise, however, their prognostic value in assessing treatment outcomes is still under investigation. A major challenge that remains with PCa clinical disease management is tumor heterogeneity. Understanding the biology and mechanisms behind the genetic and epigenetic alterations in primary and metastatic tumor, using techniques such as single cell sequencing can provide more insight into the pathophysiology of the disease. With technological advances in single-cell omics analysis and machine learning tools, together with rapid strides in imaging techniques and treatment modalities, the understanding and management of metastatic prostate cancer will continue to evolve rapidly over the next decade.

## Author Contributions

ED contributed to design of article and interpreting relevant literature. KI and FA contributed to interpreting relevent literature and developing figures. LT and RB are corresponding authors, have provided critical revisions and supervison. ML, JM and LT are experts on the topic, have supervised the manuscript preparation. All authors contributed to the article and approved the submitted version.

## Funding

Authors acknowledge the financial support by the Dutch Cancer Society (KWF) and Netherlands Organization for Scientific Research (NWO) through the PICTURES project #17915.

## Conflict of Interest

The authors declare that the research was conducted in the absence of any commercial or financial relationships that could be construed as a potential conflict of interest.

## Publisher’s Note

All claims expressed in this article are solely those of the authors and do not necessarily represent those of their affiliated organizations, or those of the publisher, the editors and the reviewers. Any product that may be evaluated in this article, or claim that may be made by its manufacturer, is not guaranteed or endorsed by the publisher.
